# A high-performance microfluidic detection platform to conduct a novel multiple-biomarker panel for ovarian cancer screening

**DOI:** 10.1039/d0ra10200h

**Published:** 2021-02-19

**Authors:** Yu Wu, Chunhua Wang, Pan Wang, Chao Wang, Yu Zhang, Lin Han

**Affiliations:** Peking University Third Hospital Haidian District Beijing 100191 China; Institute of Marine Science and Technology, Shandong University 72 Binhai Road Qingdao 266273 China hanlin@sdu.edu.cn

## Abstract

Ovarian cancer is an important leading cause of cancer-related deaths among females, and a single biomarker does not have the sensitivity and specificity required for an effective ovarian cancer screening. Herein, we investigate a high-performance microfluidic detection platform to conduct a novel panel of multiple biomarkers for the early detection of ovarian carcinoma, which include CA125, HE4, OPN, MSLN, Hsp70, CA153, AFP, IL-6, and IL-8 using a microfluidic chip. High-throughput microfluidic chips and graphene oxide-assembled substrate are used to microprint repeatable capture antibody arrays and conduct multiple biomarkers in microscale volume samples. The proposed microfluidic platform achieves an ultralow detection limit of ∼1 pg mL^−1^ and 0.01 U mL^−1^ with excellent detection selectivity and a short detection time of 30 min. The analysis of serum biomarkers in 18 ovarian cancer patients and 4 healthy persons indicates a clear subgroup sorting between the high-grade serous ovarian carcinoma, borderline, and benign tumor patients, and healthy persons. The proposed detection platform and the biomarker panel are promising to conduct an early detection of ovarian cancer.

## Abstract 2

A high-performance microfluidic detection platform is developed to conduct a novel panel of multiple biomarkers for the early detection of ovarian carcinoma, which is promising for the early detection of ovarian cancer.

## Graphical abstract

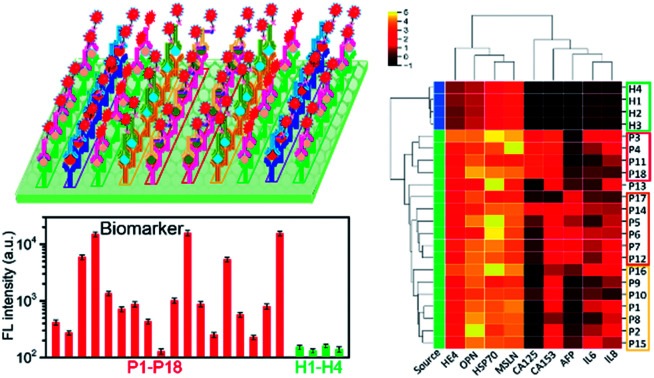

## 1. Introduction

Ovarian cancer is usually detected in the later stages, and it is the most fatal of all gynecologic malignancies with a 5 year survival rate of only 37–44%.^1,2^ As a result, it is an urgent demand to develop an effective early diagnosis approach to ensure timely treatment to essentially improve the patient outcome. CA125 and human epididymis protein 4 (HE4) are the two U.S. Food and Drug Administration-approved serum biomarkers for ovarian cancer, and HE4 plays an important role in ovarian tumorigenesis.^3^ The HE4 protein is found in elevated levels in patient serum,^4,5^ uterine fluid,^6^ and ascites.^7^ Investigation illustrates that in addition to CA125 and HE4, the expression levels of OPN,^8^ mesothelin (MSLN),^9,10^ Hsp70,^11^ and AFP^12^ are related to ovarian tumorigenesis. In the past years, serum cytokine levels have attracted particular attention as diagnostic and prognostic markers in ovarian cancer.^13,14^ Numerous cytokines are secreted by various non-hematopoietic and hematopoietic cells and are involved in immune and inflammatory responses.^12^ Some cytokines, for instance IL-6 and IL-8, seem to be secreted also by ovarian cancer cells.^13^ The interactions between the tumor and immune system and the production of cytokines by the tumor itself can result in different local and systemic levels of cytokines in cancer patients.^15^ Unfortunately, there is not a single biomarker that has a good sensitivity and specificity to conduct an efficient screening of ovarian cancer.

In the past decade, multimodal strategies have been developed with a combination of multiple biomarkers;^16–18^ for instance, the combination of CA-125 with HE4,^19^ with transvaginal sonography,^20^ with MSLN,^10^ or immune biomarkers^21^ to study the probability of improving an early ovarian cancer diagnosis. It is an urgent demand for a specific and sensitive biomarker panel to realize the noninvasive detection of ovarian cancer at an early stage. The practical applications of the detection panel in a clinic desire for a high-throughput, rapid, sensitive, and multi-marker detection platform. Biosensors integrated with the biomaterials have achieved a significant progress in the detection of biomarker *via* different sensing mechanisms, including surface-enhanced Raman spectroscopy (SERS),^22,23^ surface plasma resonance,^24,25^ electrochemical immunoassays,^26,27^ field-effect transistor,^28,29^ and implant nanosensor,^30^ which have obtained a very low detection limit and are capable of sensing a slight change in the biomarkers in samples. In order to realize the simultaneous detection of multiple biomarkers, a microfluidic technique is integrated into different detection approaches, for example, electrochemical^31^ and SERS.^32^ The combination of a microfluidic chip with a sensing module is promising to achieve the characteristics of high-throughput, low cost, high reliability, ultra sensitivity, and low reagent/sample consumption.

Here, we developed a detection platform to conduct a novel multi-biomarker panel for the early detection of ovarian carcinoma. The detection platform consisted of a GO-assembled substrate to immobilize the capture antibody, a microfluidic chip with multiple microchannels to microprint capture antibody array, and a sample loading chip with tens of microchambers. It is capable of simultaneously detecting multiple biomarkers in tens of samples. The 9-marker panel included not only conventional markers CA125, HE4, AFP, and CA153, but also OPN, MSLN, Hsp70, and inflammatory factors IL-6 and IL-8. The platform simultaneously detected all of the 9 markers in multiple samples with a detection time of 30 min and attained an ultra-low detection limit of ∼1 pg mL^−1^ and 0.01 U mL^−1^. The analysis of 9 biomarkers in samples collected from 18 ovarian patients and 4 healthy persons indicated that a combination of ovarian cancer biomarkers and inflammatory factors enabled an efficient sorting of ovarian tumors into different subgroups. Overall, the proposed multi-marker panel and the detection platform are promising in the practical applications in the early diagnosis of cancer.

## 2. Materials and methods

### 2.1 Ethics statement

The study was approved by the Peking University Third Hospital Medical Science Research Ethics Committee, China (case number: 2019 (521-02)). All the patients provided written informed consent.

### 2.2 Materials and reagents

A phosphate buffered saline (PBS) solution was purchased from Corning. CEA, AFP, and CA-153 were purchased from Fitzgerald (USA), and bovine serum albumin (BSA) was obtained from Sigma Chemical Co. (St. Louis, MO, USA). CA-125, HE4, OPN, Hsp70 and MSLN were purchased from R&D. IL-8 and IL-6 were purchased from eBioscience (USA). Streptavidin–APC was purchased from BioLegend (USA). Alexa Fluor® 488-conjugated goat anti-mouse IgG was purchased from Abcam. Silicon wafers were purchased from Meixin Electronic Technology Co., Ltd. An SU-8 2025 photoresist and developer were ordered from Bynano Co., Ltd. Treated chlorotrimethylsilane (TMCS) was purchased from Sinopharm Group Chemical Reagent Co., Ltd.

### 2.3 Microfluidic chip fabrication

The capture antibody array microprinting chip and test sample loading chip were fabricated, respectively. First, the designed pattern was transferred onto a silicon wafer *via* a standard UV photolithography process. SU-8 2025 was used to achieve microchannels of 20 μm deep in the microprinting chip. The sample loading chip possessed a sample loading chamber array, which was aligned with the repeatable antibody barcode array. After photoresist spin-coating, the silicon wafer was prebaked at 65 °C for 5 min and at 95 °C for 8 min. Then, it was exposed to UV light with appropriate energy and developed in an SU-8 developer. Finally, silicon wafers were baked at 150 °C for 40 min.

Silicon wafers with patterns were used as moulds to fabricate a PDMS chip. First, they were treated with chlorotrimethylsilane (TMCS) for 20 min in a vacuum container in order to form a monolayer to prevent the adhesion of PDMS onto the substrate. Sylgard 184 parts A and B were mixed in a ratio of 10 : 1 and loaded onto TMCS-treated silicon wafers. After the bubbles in PDMS were vacuumed, they were cured at 80 °C for 1 h. Then, the PDMS layer was peeled-off from the silicon wafer and punched to produce inlets and outlets for the antibody barcode microprinting and sample loading.

### 2.4 Material characterization

AFM characterization was conducted using a SmartSPM AFM system to perform the surface morphology of the bare substrate, capture antibody immobilized substrate, and substrate with capture antibody and captured antigen. The sample scanned area is 1 μm × 1 μm under the tapping mode at a scan rate of 1 Hz. The Raman scattering analysis of the detection process was conducted using a Renishaw inVia Raman microscope at room temperature with a 532 nm line of an Ar ion laser as an excitation source. In order to characterize the detection process, the bare substrate, capture antibody-immobilized substrate, and substrate with capture antibody and captured antigen were characterized. Because of the weak Raman signal of the detection target, silver nanoparticles were used to achieve surface-enhanced Raman scattering.

### 2.5 Biomarker sample and clinical sample preparations

The recombinant protein was retrieved from a refrigerator and was diluted with 1% BSA at different concentrations: 0.1 U mL^−1^, 1 U mL^−1^, 10 U mL^−1^, and 100 U mL^−1^ for CA125 and CA153; 0.1 pg mL^−1^, 1 pg mL^−1^, 10 pg mL^−1^, and 100 pg mL^−1^ for OPN; 1, 10, 100, and 1000 ng mL^−1^ for MSLN; 0.1 ng mL^−1^, 1 ng mL^−1^, 10 ng mL^−1^, and 100 ng mL^−1^ for Hsp70; 1 pg mL^−1^, 10 pg mL^−1^, 100 pg mL^−1^, and 1000 pg mL^−1^ for AFP, IL-6 and IL-8.

Clinical blood samples were collected in non-anticoagulant tubes and placed in a sterile environment at room temperature for 3–5 h. The upper layer of the pale-yellow liquid was collected, which was then centrifuged at 3000 RPM at 4 °C for 10 min. The upper serum liquid was collected in an EP tube and stored at −80 °C, and was then ready for use anytime.

To detect the biomarkers in the samples, the samples were retrieved from the refrigerator and thawed. Then, 2 μL of each sample was collected using a pipette and loaded into the detection chamber on the sample loading chip. After incubation for 10 min, the chip was cleaned using PBS and clean water, and then, the detection antibodies were loaded.

### 2.6 Detection antibody complex preparation

Detection antibodies were mixed at a concentration of 1 mg mL^−1^ in 1% BSA and a total volume of 300 μL achieved. 3 μL of fluorescence-labeled streptavidin was loaded into the mixed detection antibodies labeled with biotin and incubated for 10 min to form a complex of detection antibodies.

### 2.7 Fluorescence signal scanning

After the target antigen was captured and the detection antibody conjugated, the chip was cleaned thoroughly, and its fluorescence intensity was scanned using a laser scanner GenePix 4400 with excited lasers of 488 nm and 635 nm, PMT of 350–500, power of 90, and resolution of 2.5–10 μm. Then, the fluorescence pattern was processed using the GenePix software.

## 3. Results and discussions

### 3.1 Detection chip of multiple biomarkers

**Fig. 1:**
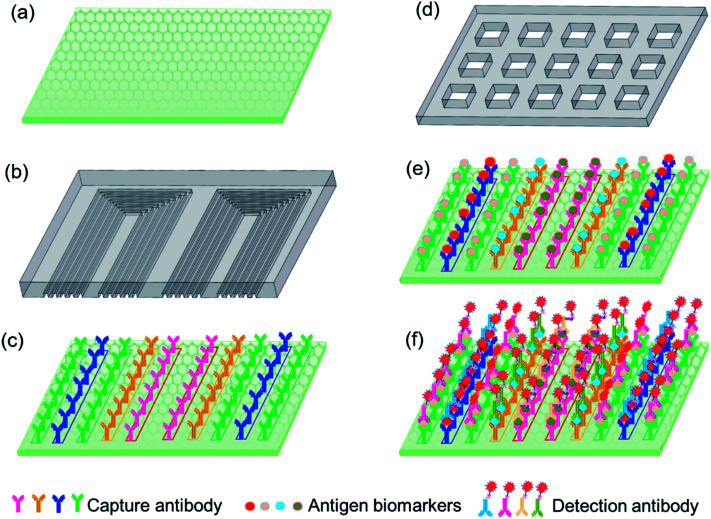
(a) Detection substrate, (b) microprinting chip, (c) capture antibody barcode microprinted on the substrate, (d) sample loading chip, (e) biomarker antigens captured by the antibody barcode, and (f) fluorescence-labeled detection antibody specifically conjugated with biomarkers.

As shown in Fig. 1a, the detection substrate is modified with graphene oxide to immobilize the capture antibody protein *via* simple π–π stacking, which essentially simplifies the operation process compared with the commonly used covalent bonding assisted by 3-(3-dimethylamino propyl)-carbodiimide (EDC) or *N*-hydroxysulfosuccinimide sodium salt (NHS). A microfluidic chip is designed with multiple parallel microchannels, as shown in Fig. 1b, which is to realize the local microprinting of the capture antibody array on the detection substrate, and form different antibody barcodes in individual microchannels, as shown in Fig. 1c. The microprinting chip only consumed 2 μL of each antibody to form multiple repeatable U-shaped detection units along the whole chip, which greatly reduced the antibody expense compared to the traditional ELISA method. In order to avoid the non-adsorption onto graphene oxide, after the capture antibody barcode was microprinted onto the graphene oxide substrate, the microfluidic chip used for microprinting was peeled off in 3% BSA to aid in the area outside capture antibody barcode to be blocked by BSA. The sample loading chip shown in Fig. 1d is fabricated with microwells, and each microwell covers a whole array of the capture antibody barcode so that all of the ten biomarkers in the test samples are captured by the immobilized antibodies. Then, the biomarker antigen is incubated with the immobilized antibody for 10 min, and finally, the fluorescence-labeled detection is specifically conjugated with the antigen with a reaction time of 10 min, as shown in Fig. 1e and f. The fluorescence intensity was utilized to quantify the concentrations of different biomarkers in the loaded samples. The total detection time was 30 min, including the incubation time between the capture antibody and antigen biomarker, the detection antibody and biomarker, and the washing time and the fluorescence signal scanning time.

### 3.2 Characterization of sensing substrate

**Fig. 2:**
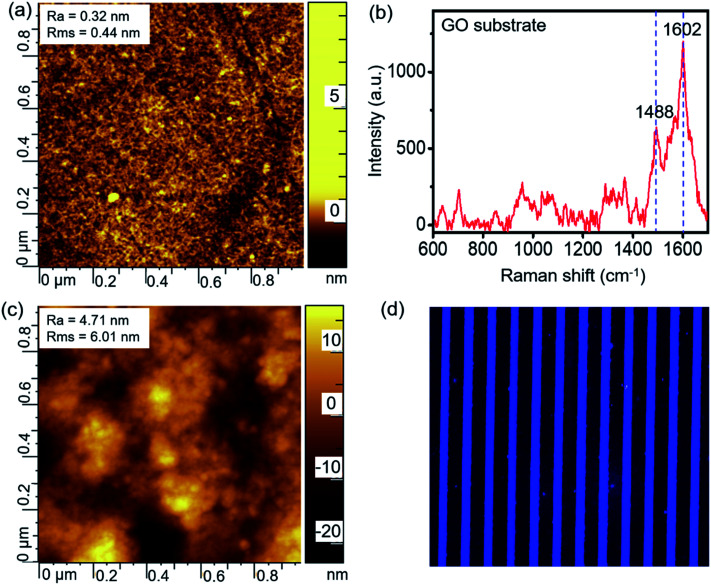
(a) AFM and (b) Raman spectrum characterizations of the GO substrate, and (c) AFM and (d) fluorescence images of the capture antibodies immobilized onto the GO substrate.

The GO-assembled glass substrate was used together with microfluidic channels to conduct the multiple biomarker detection. The substrate was modified by APTES, following which the graphene oxide nanomaterial was assembled. AFM and Raman spectra were recorded to characterize the detection substrate. As indicated by the AFM image in Fig. 2a, the surface is smooth after the graphene oxide assembled with a roughness of 0.32 nm, and the Raman spectrum in Fig. 2b confirms the successful assembling of graphene oxide with representative peaks at 1488 and 1602 cm^−1^. GO on the glass substrate provided a nano-scale rough surface, and the large surface area benefited the high-efficient immobilization of the capture antibody. The capture antibody was immobilized onto the substrate *via* the interactions with graphene oxide, which enabled the subsequent detection of biomarkers. The AFM image in Fig. 2c shows a slightly larger roughness after the immobilization of the capture antibody compared to the GO substrate. In order to confirm the successful immobilization of the capture antibody onto the GO substrate, the fluorescence-labeled antibody was microprinted and incubated on the substrate for 3 h. Then, the substrate is washed thoroughly and scanned using the laser scanner to obtain the fluorescence image, as shown in Fig. 2d. The uniform fluorescence signal on all the microprinting locations indicated a full and uniform recovery of antibodies on the GO substrate.

### 3.3 Sensitivity and specificity of the ovarian cancer biomarker detection

**Fig. 3:**
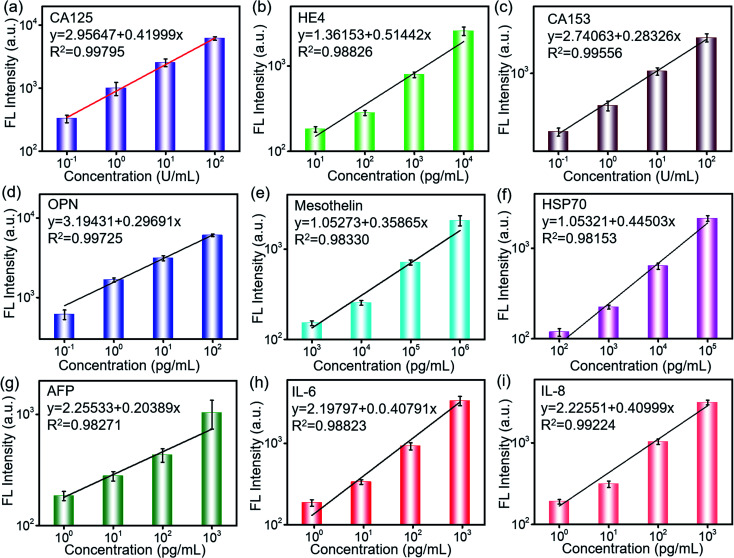
Fluorescence dependence on the concentration of biomarkers: (a) CA125, (b) HE4, (c) CA153, (d) OPN, (e) MLSN, (f) Hsp70, (g) AFP, (h) IL-6, and (i) IL-8.

The sensitivity and specificity of the detection are important characteristics for the cancer biomarker detection. 2 μL of the biomarker antigen sample with different concentrations was loaded *via* a loading chip, which was aligned with the antibody barcode substrate and reacted with the capture antibody for 10 min. The fluorescence-labeled detection antibody was loaded and incubated for 10 min. After complete washing, the fluorescence scanning was conducted. As shown in Fig. 3, the fluorescence intensity increases with the increase in the biomarker concentration and presents a linear relationship in the log–log scale. Based on these quantitative equations, the concentrations of the biomarkers were derived during the clinical sample detection. The derived detection limit at 3S/N reached 1 pg mL^−1^ and 0.01 U mL^−1^ according to their different units. The detection limit was much lower than that of the clinical cutoff values for all of the biomarkers. Furthermore, this was beneficial to conduct early detection because the biomarkers may experience a slight change in the secretion level. The sensitive detection of the biomarkers is contributed not only by the efficient capture of the GO substrate but also by the high-performance of the laser scanner. The fluorescent molecule was excited by a laser, and the excited weak fluorescence signal was amplified by a photomultiplier tube (PMT). As a result, the performances of the excitation laser, PMT, optical system, and signal processing were also important to realize the sensitive detection. In addition, the error bars from the three tests indicated its high repeatability and reliability, which were very important in the practical applications.

**Fig. 4:**
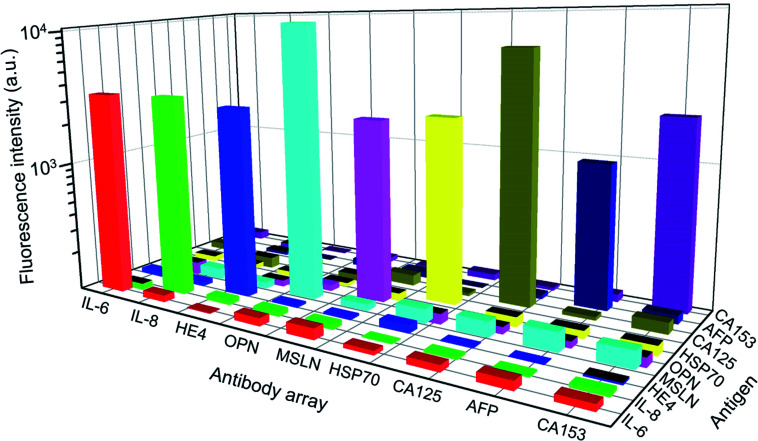
Detection specificity of the biomarker panel. The used concentrations of the biomarkers are as follows: CA125 and CA153: 100 U mL^−1^; IL-6, IL-8, and AFP: 1 ng mL; OPN: 100 pg mL; HE4: 10 ng mL; Hsp70: 100 ng mL; and MSLN: 1 μg mL^−1^.

The detection specificity of the biomarkers is extremely important as they contain certain amounts of biomolecules in the serum. Fig. 4 shows that all of the antibodies have a specific reaction with corresponding biomarkers in the detection panel, and a paired antigen–antibody presents much higher fluorescence intensity than the mismatched pairs. Furthermore, the fluorescence signal of the mismatched pairs was close to the background fluorescence. During all of the tests, each data point was repeated for three times, and the error bars represented the difference between tests, which was negligible compared to the detected signals. The excellent specificity of the proposed panel enabled the high-performance detection of multiple biomarkers in the clinical serum samples.

**Fig. 5:**
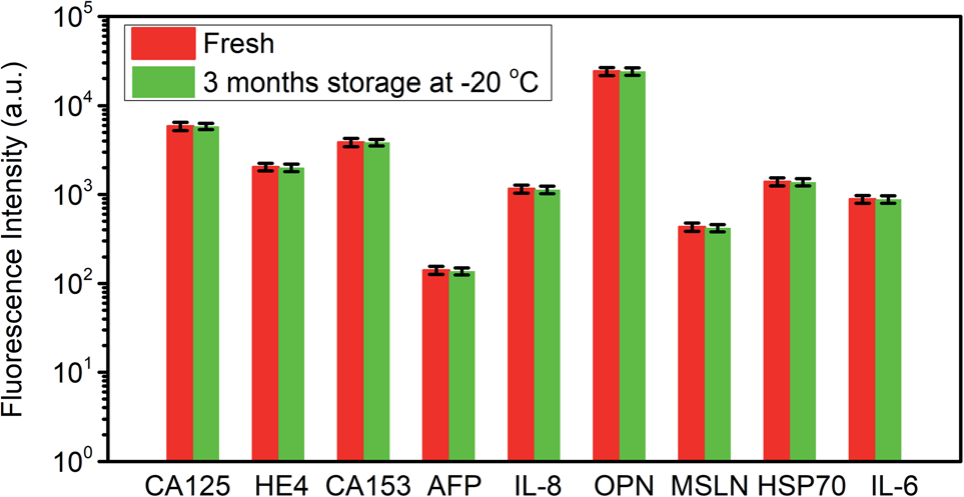
The stability of the detection chip after 3 month storage at −20 °C.

In order to evaluate the stability of the antibodies immobilized on the substrate, one chip prepared 3 months ago was utilized to repeat the detection of biomarkers, and the prepared chip was stored at −20 °C in the refrigerator after the immobilization of antibodies. The detected signal is compared with the data obtained 3 months ago, as shown in Fig. 5. The fluorescence intensity variation is less than 5%, which indicated an excellent stability of the proposed detection chip.

### 3.4 Real sample detection and analysis

**Fig. 6:**
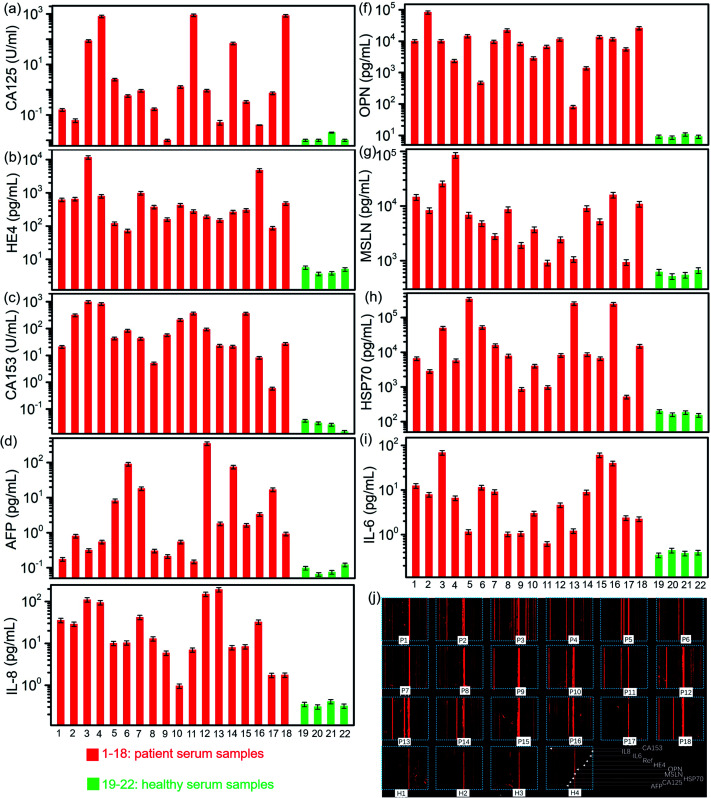
Detected concentrations of biomarkers in ovarian cancer patients and healthy persons: (a) CA125, (b) HE4, (c) CA153, (d) OPN, (e) IL-8, (f) OPN, (g) MSLN, (h) Hsp70, and (i) IL-6. (j) Fluorescence image of the detected samples.

We used the well-developed microfluidic chip to detect the biomarkers in the clinical serum samples, which included 18 ovarian cancer patients and 4 healthy persons. All the 9 biomarkers in 22 samples were conducted on one chip. Their detected concentrations are derived from the quantitative equations, as shown in Fig. 3 and 6. All the 9 biomarkers presented a much higher expression level in most patient sera than those in healthy sera, particularly HE4, OPN, MSLN, Hsp70, and CA153. The level of OPN, MSLN and Hsp70 appeared to be very high even at an early stage of the ovarian tumor, while HE4 and CA153 presented a relatively high expression level. The level of CA125 was not high in some patients' sera, particularly in the early stage, which indicated its low diagnostic sensitivity as a biomarker in the ovarian cancer early detection. The inflammatory factors, IL-6 and IL-8, were produced by numerous hemopoietic and nonhemopoietic cells, which regulated and mediated inflammation, hemopoiesis, and immunity. Previous investigation found that the ovarian cell lines and primary ovarian cancer cell cultures seem to produce tumor-promoting IL-6 and IL-8. Serum detection indicates that both have higher secretion levels in ovarian cancer patients than those in healthy persons, as shown in Fig. 6a–i, in which the concentrations of biomarkers are derived from the fluorescence image shown in Fig. 6j. As a result, the combined screening of these biomarkers was probably an efficient way to conduct the early diagnosis of ovarian cancer. The detection chip demonstrated a low detection limit, fast detection time, large linear regime and capability to conduct multiple biomarker detection. Its major performance is listed together with a few representative detection methods in Table 1.

**Table 1:** Main detection performance in representative methods

Detection method	Biomarkers	Detection limit	Detection time	Linear region	Ref.
Electrochemical impedance spectroscopy	CA125	0.923 ng μL^−1^	∼20 min	0.92 pg μL^−1^ to 15.2 ng μL^−1^	27
Optical nanosensor	HE4	1 nM	∼60 min	NA	28
Microfluidic chip electrophoresis	CA125, CEA, AFP	0.1–0.2 pg mL^−1^	∼80 min	0.01–30 ng mL^−1^	33
Microfluidic chip RCA	IL-8	0.84 pM	∼3 h	7.5–120 pg mL^−1^	34
Electrochemiluminescence immunosensor	AFP	10 fg mL^−1^	∼1 h	0.1 pg mL^−1^ to 200 ng mL^−1^	35
Our work	9 biomarkers	1 pg mL^−1^, 0.01 U mL^−1^	∼30 min	1–10^3^ pg mL^−1^ (HE4), 0.1–100 U mL^−1^ (CA125, CA153)	This work

**Fig. 7:**
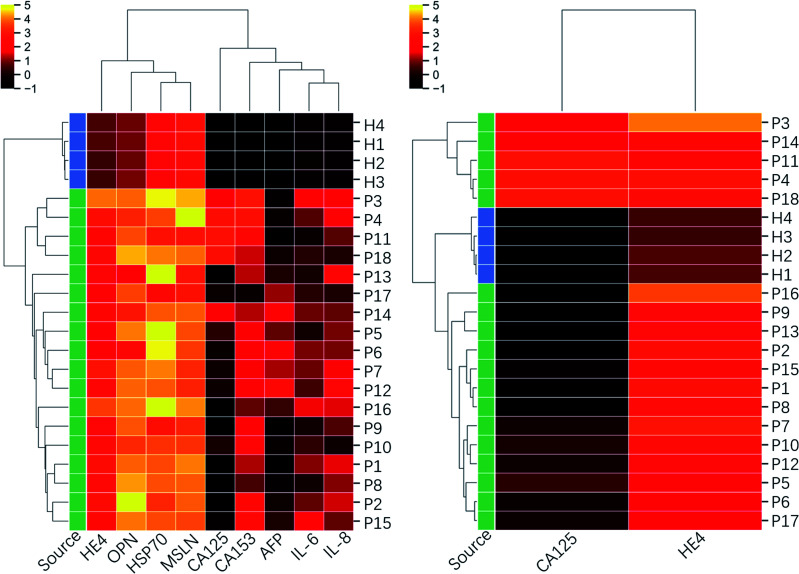
Clustering heatmap based on the inflammatory factors integrated 9-biomarker panel and CA125–HE4 biomarker panel. The dataset used for the clustering is in log scale.

In order to further explore the screening efficiency of the proposed biomarker panel, the tested clinic samples were sorted using the Hierarchical Clustering method based on the 9-marker panel and the traditional 2-marker panel (CA125 and HE4). It initialized each sample in the dataset as a cluster, found the nearest two clusters, merged them, and repeated the process until the preset number of clusters. For dataset covering a few orders of magnitude, it is usually standardized in a log scale, as used in the following clustering analysis. The clinical diagnosis results are provided in Table 2. The clustermap in Fig. 7 presents that 4 healthy samples are sorted out of patients' samples, and the patients' samples are categorized into 3 subgroups based on the 9-marker panel. The first group (P3, P4, P11, and P18) consisted of all the samples from the high-grade serous ovarian carcinoma patients, which demonstrated a bad prognosis. The second group (P5, P6, P7, P12, P14, P17 except P12) consisted of all the samples from the benign patients, including endometriosis cyst, mucinous cystadenoma, ovarian fibrothecoma, tuberculosis of pelvic and abdominal cavity, which demonstrated a good prognosis. In addition, the third group (P1, P2, P8, P9, P15, and P16) consisted of samples from both benign and borderline tumor patients, including borderline seromucinous cystadenoma, borderline myxoma, endometrioid adenofibroma, serous cystadenoma, and ovarian endometriosis cyst, whose prognosis was between groups 1 and 2. Among the third group, P10 was diagnosed as the high-grade serous ovarian carcinoma by the comprehensive judgment on the symptom, ultrasound and nuclear magnetic resonance image, but the proposed chip did not detect high levels of CA125. P13 was clustered out of all the third groups, but the clinical diagnosis indicated the type of fibrothecoma. The analysis results showed that only two patients out of 22 persons were not consistent with the clinical diagnosis, which indicated a success prediction rate of ∼91%. However, the clustermap based on only CA125 and HE4 could not clearly recognize different subgroups, even though the healthy group were sorted correctly. The analysis results indicated that the proposed biomarker panel was promising in the early detection of ovarian cancers.

**Table 2:** Clinical diagnosis results of patients

Sample source #	Clinical diagnosis
P3, P4, P10, P11, P18	High-grade serous ovarian carcinoma
P1	Endometriosis cyst with serous cystadenoma
P2	Leiomyosarcoma
P5	Borderline seromucinous cystadenoma
P6	Borderline myxoma is not enough for the diagnosis of mucinous adenocarcinoma, but the growth is active and the risk of recurrence is high
P7, P12	Endometrioid adenofibroma
P8	Mucinous cystadenoma
P9, P13, P16	Fibrothecoma
P14	Serous cystadenoma
P15	No tumor visible, pelvic and abdominal tuberculosis
P17	Ovarian endometriosis cyst

## 4. Discussions and conclusions

In this study, the proposed microfluidic platform presented advantages of high-throughput, rapid, and sensitive characteristics to detect the multiple biomarkers in tens of samples. The platform provided a valuable tool for the detection of biomarkers in the early stage of ovarian tumor because of its high detection sensitivity. The tests of clinical samples demonstrated that the ovarian cancer serum samples not only had higher level of conventional CA125 and HE4 markers, but also OPN, MSLN, Hsp70, CA153, and inflammatory factors IL6 and IL8 compared to the healthy persons. Based on this integrative biomarker panel, the patients in different cancer subgroups were recognized clearly by the Hierarchical Clustering, which is a traditional sorting method. The presence of biomarkers in high levels at an early stage may be a good option to be combined with CA125 to realize the early diagnosis of ovarian cancer. However, to improve the diagnostic sensitivity and specificity of ovarian cancers, more clinical samples in different subtypes are needed because the collected samples in this study are limited and not enough. In summary, the proposed study offers a high-performance detection platform, which is universal for the detection of protein biomarkers, and a promising multi-marker panel for the early diagnosis of ovarian cancer.

## Author contributions

Yu Wu: investigation, validation, formal analysis, project administration resources, writing-original draft; Chunhua Wang: methodology, validation; Chao Wang: methodology, software; Pan Wang: investigation; Yu Zhang: conceptualization, methodology; Lin Han: data curation, supervision, funding acquisition, writing – review & editing.

## Conflicts of interest

All of the authors declare no conflicts of interest in this work.

## Supplementary Material
